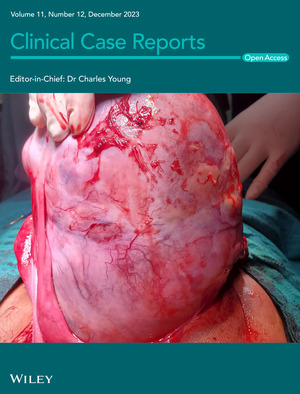# Cover Image

**DOI:** 10.1002/ccr3.8387

**Published:** 2023-12-25

**Authors:** Aashish Poudel, Prajwal Sedain, Biraj Pokhrel, Aakash Sapkota, Anita Chamlagain, Nisha Sharma, Sanyukta Rajbhandary, Bishal Khaniya, Neebha Ojha

## Abstract

The cover image is based on the Case Report *A large yolk sac malignancy in a girl, an uncommon yet challenging ovarian tumor: A case report* by Aashish Poudel et al., https://doi.org/10.1002/ccr3.8335